# Retailer’s Dual Role in Digital Marketplaces: Reference Architectures for Retail Information Systems

**DOI:** 10.1007/s42979-022-01098-w

**Published:** 2022-03-31

**Authors:** Tobias Wulfert, Reinhard Schütte

**Affiliations:** Institute for Computer Science and Business Information Systems, University of Duisburg-Essen, Universitätsstraße 2, 45141 Essen, Germany

**Keywords:** Digital marketplace, Architectural pattern, Retail information system, Electronic commerce, Design science research

## Abstract

Digital marketplaces have entered the retail sector and have proven to be a successful business model compared to traditional retailing. Established retailers are increasingly launching digital marketplaces as well as participating in marketplaces of pure online companies. Retailers transforming to digital marketplaces orchestrate formerly independent markets and enable retail transactions between participants while simultaneously selling articles from their own assortment to customers in the digital marketplace (dual role). A retailer’s dual role must be supported by retail information systems. However, this support is not explicitly represented in existing reference architectures for retail information systems. Thus, we propose to develop a reference architecture for retail information systems that facilitates the orchestration of supply- and demand-side participants, selling their own articles, and providing innovation platform services. We apply a design science research approach and present nine architectural requirements that a reference architecture for a multi-sided market business model in retail needs to fulfill (dual role, additional participants, affiliation, matchmaking, variety of services, innovation services, smart services, aggregated assortment, and boundary resources) from the rigor cycle. From the first design iteration, we propose four exemplary, conceptual architectural patterns as a solution for the requirements (matchmaking for participants, innovation platform services, boundary resources, and aggregated assortment). These patterns can form a conceptual reference architecture that guides the design and implementation of information systems.

## Introduction

Catalyzed by the implications of the Covid-19 pandemic, electronic commerce (e-commerce) revenue has increased worldwide and across sectors by 11% annually since 2019 and is expected to reach USD 3,300 billion by 2024 [96]. In Europe, e-commerce accounted for EUR 374 billion in total in 2019. As customers are willing to shop at brick-and-mortar retailers only if they feel safe, they increasingly tend to shop for groceries, apparel, jewelry, etc., online and aim for digital end-to-end customer journeys [19, 36, 82]. Additional government mandates that closed brick-and-mortar shops forced retailers to (hastily) establish additional online sales channels and transform their value proposition in favor of digital marketplaces [36, 86]. Sales via the marketplace accounted for more than 60% of Amazon’s revenue in the fiscal year 2019 [125]. The pandemic has even amplified digital transformation in retail and wholesale that previously neglected necessary digitalization endeavors [100].

In addition to the possibility of establishing electronic shops, retailers and wholesalers may participate in existing or create their own digital marketplaces [55, 106, 115]. While (Electronic) shops act as resellers in a single market, but digital marketplaces connect previously independent markets, match individual participants from the multiple market sides, and enable (retail) transactions between the parties [57]. Digital marketplaces orchestrate multiple markets and simplify interactions with suppliers, logistic service providers, market researchers, and other actors [21, 59]. These marketplaces focus on monetization of the matchmaking instead of selling articles with higher margins [27, 45, 70]. The orchestration causes (merely indirect) network effects for market participants [104], and marketplace owners implement asymmetric pricing mechanisms to monetize the matchmaking [7, 94]. The revenue generated by a digital marketplace and its surrounding ecosystem is up to three times higher compared to that of other business models [35]. Although traditional retail companies act as market intermediaries that offer manufacturers’ products to customers (reseller mode), the development of digital marketplaces for the implementation of multi-sided markets (as a generalization of two-sided markets) has not been driven by retail, but usually by technology companies. Examples of the tremendous success of digital marketplaces include Amazon, Alibaba, and eBay, which orchestrate multiple market sides [99]. It seems unusual that although retailers are a significant factor in any economy and have traditionally linked markets (e.g., producer and consumer markets) for a long time [76], the expansion to the orchestration of multiple market sides with digital marketplaces was not driven by retailers. Thus far, only a few large retail companies have established their own digital marketplaces (e.g., Walmart and REWE). As digital marketplaces often form the preferred touchpoint for many customers, and marketplace owners exclude manufacturers from customers, retailers need to establish their own marketplaces [82, 96].

If the owner of a digital marketplace behaves neutrally [71, 117], the company does not gain ownership of the traded articles (in contrast to e-shops) at any point during the transaction [56]. A digital marketplace facilitates retail transactions between ecosystem participants by providing interfaces for the interaction [120]. In contrast, competitive marketplace owners possess a dual role and offer their own articles in the digital marketplace [71, 107]. They may compete with other supply-side participants offering similar articles. This dual role as marketplace owner and competitor selling their own articles in the digital marketplace creates additional requirements for a retail information system [126]. Although the importance of multi-sided market business models has seemed to grow, existing literature focuses only on the adaptation of business models and the respective tools for modeling them [45, 92]. The consequences of the retailer’s dual role for the underlying retail information system-the digital infrastructure for e-commerce-supporting the specifics of the e-commerce and intermediary business model of multi-sided markets are rarely considered [10, 126]. A reference architecture may help to decrease setup time for the digital infrastructure supporting digital marketplaces and standardize processes and interfaces [4]. Reference architectures also facilitate the introduction of new processes and technologies in e-commerce and allow for better value co-creation within the surrounding ecosystem because of well-documented boundary resources [38, 91]. This standardization may ease participation in the digital marketplace and increase network effects [38, 104]. Although domain-specific reference architectures for the retail sector, such as the h-model [16] and the ARTS model [6, 87], do exist, literature dealing with domain-specific reference architectures for e-commerce in general [10] and a retailer’s dual role in digital marketplaces in particular is sparse according to our research. Thus, we address this research gap by deriving architectural requirements (ARs) for a retail information system caused by the transformation of a retailer in reseller mode to a marketplace owner and present focal architectural patterns that address these requirements. For the analysis of architectural requirements for digital marketplaces and the development of architectural patterns, the focus is on the combination of three aspects: the orchestration of formerly independent markets in the sense of multi-sided markets [7, 23, 60, 94], competitive marketplace owners selling their own articles [56, 71], and the establishment of digital platforms from a technical software perspective [50, 111]. The architectural patterns include aspects of technology platforms that center the marketplace owner’s retail information system as a key technological artifact upon which further modules can be developed [50, 118]. To derive architectural requirements, we follow a design science research approach [89] with the architectural patterns as artifacts [79]. We use ArchiMate as the language for modeling enterprise architectures to formally present the architectural patterns in a “unified, unambiguous, and widely understood domain terminology” [85]. For the presentation of the architectural patterns, we focus on the business and application layers.

This research paper is an extended version of a paper presented at the 23rd International Conference on Enterprise Information Systems. For this special issue of the *SN Computer science* we have modified the introduction, added three architectural requirements and an architectural pattern, analyzed reference architectures in literature for the fulfillment of the requirements and patterns, improved the discussion section, and streamlined the concepts used throughout the text. The remainder of the paper is structured as follows. First, we introduce related literature concerning architectural patterns in information systems (IS) architecture and digital marketplaces in e-commerce and present nine architectural requirements. Second, we outline our research approach for deriving architectural patterns. Third, we present three exemplary architectural patterns and elicit additional architectural considerations for digital marketplaces. Finally, we discuss the architectural patterns, present findings from a preliminary reference architecture analysis, and summarize the results.

## Related Literature

### IS Architecture and Patterns

Retail information systems include all application systems that are used to support operational tasks in retail. The retail information system supports the execution of the main trading functions and related tasks that bridge the discrepancies in the streams between manufacturers and customers in real goods (goods, services; returns), nominal goods (money, credits), and information across space, time, quantity, and quality [14, 76]. Indeed, Retail information system support operational-dispositive, business-administrative, controlling, and corporate planning tasks [16]. In addition to merchandise management (merchandise planning, logistics, and settlement processes), retail information systems also support business intelligence and necessary corporate-administrative tasks in an integrative manner [98]. Facilitated by the ongoing digitalization of the retail sector, the bridging functions increasingly address digital product and price information and adaptations in payment, logistics, and distribution processes [16, 100]. In e-commerce, transactions are carried out digitally to some degree [75]. Thus, they build digital infrastructures for executing the trading function in online and offline environments. Reference architectures are reference models (scripts) that serve as architectural templates for the design of a specific architecture in the context of a specific company [15, 91]. They are developed using a specific grammar and guided by an implementation method (i.e., a procedure model) [91]. An information systems architecture “is a set of high-level models which complements the business plan in IT-related matters and serves as a tool for information systems planning and a blueprint for information systems plan implementation” [119]. Information systems architectures comprise a high-level sketch of the system and application architecture of a specific company and part of its application architecture [64].

Concrete information systems architectures deal with one particular company. However, reference architectures abstract from the company’s peculiarities, therefore enabling the reuse of architecture components, providing an agreed-upon set of concepts and architectural patterns, and communicating fixed viewpoints [52]. The development of a reference architecture is often inspired by concrete architectures or other artifacts and thus has a “descriptive nature” [48]. Developing a reference architecture based solely on existing research in a prescriptive manner allows the creation of “a futuristic view of a class of systems” [48]. Reference architectures focus on clarification of innovative patterns and aim to convince domain architects of the architecture qualities. Consequently, concrete systems can be developed according to this research-based architecture [5]. Reference architectures are applied either to standardize existing systems to ensure interoperability or to facilitate the design and improve the quality of a concrete architecture with architectural guidelines [3, 5]. They can be used as a starting point for company-specific models to reduce the effort of creating them through reuse of established artifacts and constructs [121]. A domain-specific reference architecture is a reference model at a high level of abstraction that provides a view of the essential areas of a domain (e.g., AUTOSAR) without having to consist of complete process and data models [48, 85]. A reference architecture is the mapping of process and data models’ functionality onto system modules [47]. Domain-specific information systems reference architectures for the retail sector offer a high-level view on architecture components and business functions.

**Fig. 1:**
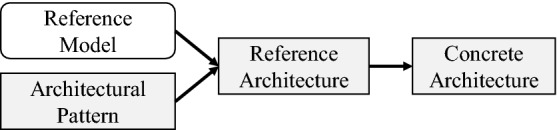
Relation between architecture types [125]

A reference architecture consists of several architectural patterns [28, 105]. These patterns are defined as a “named collection of architectural design decisions that are applicable to a recurring design problem” [108]. The patterns are reusable solutions to common architectural problems within a given domain [105]. In addition, architectural patterns are often parameterized so that they can be applied to specific issues in different organizational contexts [108]. The relation between a reference model and architectural patterns as building blocks of the reference architecture and its manifestation in a concrete architecture for a company are illustrated in Fig. 1.

### Digital Marketplaces

Introducing the idea of digital marketplaces in e-commerce, we draw on the concept of two-sided markets [7, 57]. Digital marketplaces match two or more previously distinct markets and exploit direct and indirect network effects to further propel the digital marketplace (e.g., one side of the market subsidizing the other [8, 94]). Digital marketplaces have predominantly operated in the B2C and C2C contexts, but they are starting to be used for B2B transactions more frequently [78]. Although the concept of marketplaces is also present in brick-and-mortar retail with shopping malls or variants of trading such as agency trade [1] and commission business [84], the network effects for participants (lower transaction costs for search and initiation) and economies of scale for market owners (the marginal costs for adding another supplier or article are almost zero) are even stronger in e-commerce. Digital marketplaces form the center of digital business ecosystems in e-commerce [101].

**Fig. 2:**
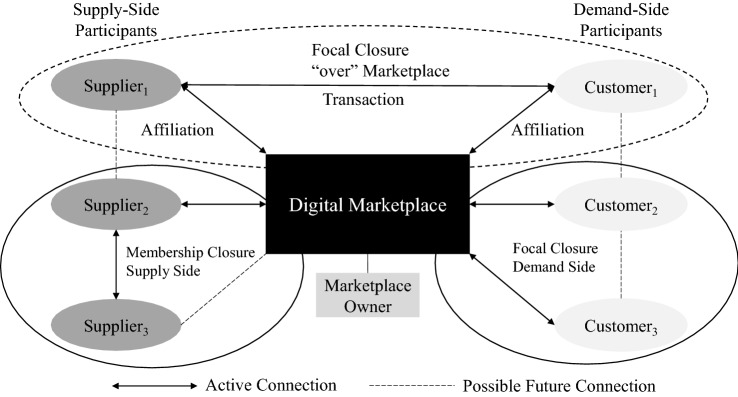
Triadic closure in marketplace-centered digital business ecosystems

With direct (or same-side) network effects, the value of a digital business ecosystem for a demand- or a supply-side participant increases with the size of the network on the same side. This is due to the higher potential of exchange (e.g., of a product review or knowledge) between these actors [41, 81]. An indirect (or cross-side) network effect in a digital business ecosystem arises if the benefit to actors on the supply side depends on the number of participants on the demand side and vice versa (e.g., the supply side of the digital business ecosystem subsidizing the demand side) [8, 94]. A prerequisite for indirect network effects is the presence of cross-group-side network effects in both directions [57, 104]. Applying graph theory, network effects can be described as triadic closures (focal or membership) in social affiliation networks on one (direct, black circles) or multiple sides (indirect, dashed circles) in a digital business ecosystem with a focal digital marketplace (Fig. 2). The digital marketplace is the foundation for orchestrating multiple market sides [37]. The ecosystem dynamically evolves as actors join or depart, while creating new or interrupting previous stable triadic closures [126]. The participants in digital business ecosystems are interdependent. They cooperate to achieve common objectives, usually competing for scarce resources at the same time [30]. Supply-side participants offering similar merchandise compete for customers from the demand side.

Establishing a digital marketplace poses additional requirements for a retailer’s IS. The further elicitation of digital marketplaces is used to derive and is structured by nine architectural requirements resulting from a retailer’s dual role that are summarized in Table 1.

**Table 1:** Architectural requirements of a retailer’s dual role in digital marketplaces

#	Architectural requirement	Description
1	Retailer’s dual role	A retailer as a marketplace owner can also behave competitively with participants [59, 71]
2	Additional types of participants	The different participants need to be represented adequately in terms of master data, and matching records are required [21]
3	Affiliation with the marketplace	Participants require an affiliation to the marketplace to conduct transactions [57]
4	Matching as a core value proposition	Matching between individual participants of the formerly independent market sides must be enabled [83]
5	Diversity of services	Services offered by the marketplace owner differ in type, scope, and coverage (modularity) and can be added as the digital marketplace evolves (pluggability) [126]
6	Innovation platform services	Technical capabilities that enable the creation of innovative solutions by developers [111]
7	Smart service provision	Support the management of smart products and provision of smart services [18]
8	Aggregated assortment	Digitally aggregate the assortment of several supply-side participants [109]
9	Sophisticated boundary resources	Provide boundary resources for different participants to connect to the marketplace [33, 38]

#### Architectural Requirement 1: Retailer’s Dual Role

**Fig. 3:**
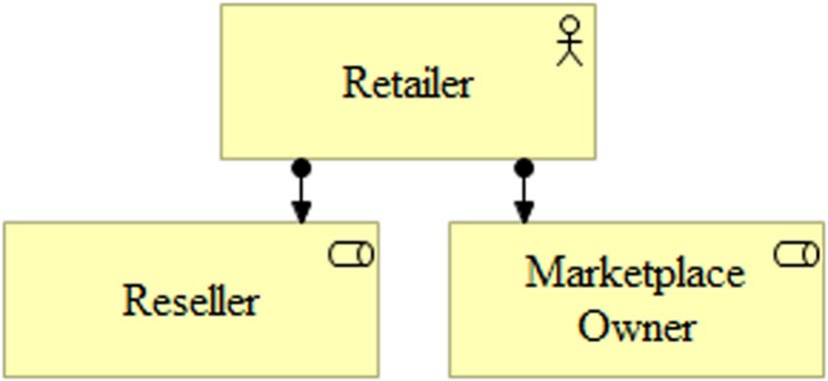
Retailer’s dual role [125]

In addition to taking a neutral role by merely facilitating the matchmaking, the marketplace owner can behave competitively with supply-side participants offering its own articles to demand-side participants [126]. Hänninen et al. distinguish pure multi-sided digital platform business models that merely facilitate matches between the supply and demand sides (e.g., eBay, Alibaba, and Rakuten) and multi-sided market business models that extend their own range of articles with independent suppliers and offer further services to them (e.g., Amazon) [59, 77]. The focus of this research paper is on the retailer’s dual role as simultaneously a marketplace owner and a reseller that competes with other supply-side participants (Fig. 3).

In addition, marketplaces can be established based existing brick-and-mortar stores or electronic shops as additional sales or procurement channel [71]. The marketplace owner can be either one (e.g., Walmart Marketplace) or a conglomerate of the participants (e.g., Opodo) or even an independent third party (e.g., eBay) [117]. *Requirement:* The reference architecture should support the orchestration of the market sides [92] and traditional bridging functions with related tasks [76, 84].

#### Architectural Requirement 2: Additional Types of Participants

**Fig. 4:**
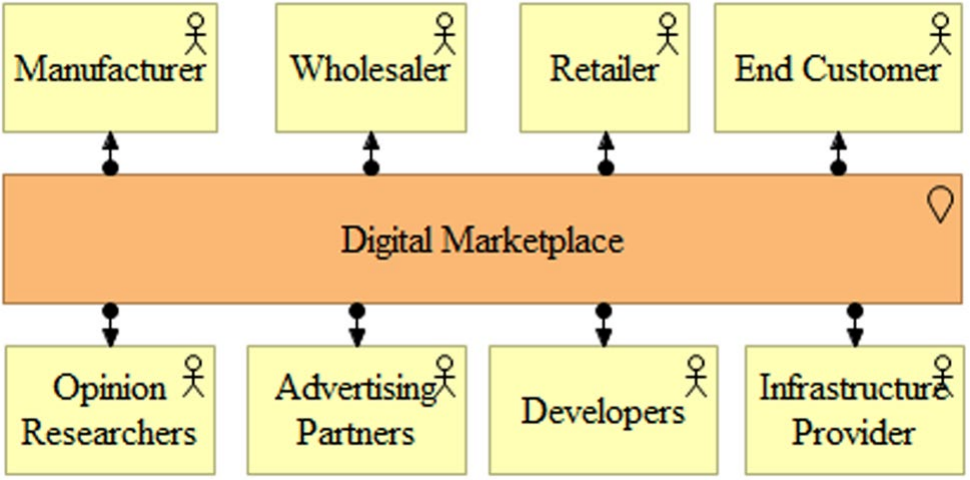
Digital marketplace participants [125]

For the development of architectural patterns, we focus on the two most important market sides, suppliers (manufacturers, wholesalers, and retailers) and customers (end customers and retailers). In general, we describe a two-sided market as a specific manifestation of a multi-sided market in e-commerce [57]. Moreover, we also integrate third-party developers and infrastructure providers to support the innovation platform perspective [50, 111]. Possible additional market sides are, among others, advertising partners, logistics service providers, or opinion research agencies. Digital marketplaces differ from the traditional value chain of (offline) retailers and electronic shops in that digital marketplaces match manufacturers on the supply side with end customers on the demand side. Retailers and wholesalers may interact with a digital marketplace as a supplier or may demand articles from the digital marketplace that is controlled by the marketplace owner (Fig. 4). The digital marketplace is modeled as a location at which the matching and (parts of) the transaction are executed [54, 112]. *Requirement:* The different participants within a digital marketplace should be represented adequately in terms of master data, and records need to ensure that transactions between the participants can be tracked to optimize future matchmaking.

#### Architectural Requirement 3: Affiliation with the Marketplace

In the offline environment, retailers try to establish relations with their customers by offering, for example, optional loyalty cards or apps [58, 97]. Hagiu and Wright [57] contend that digital marketplace participants always require some affiliation with the marketplace. However, the way in which the digital marketplace participants must be affiliated is not further defined and can be interpreted differently (e.g., contract, membership, and cookies) [57]. The affiliation is important to improve the likelihood and quality of the matching, as the affiliation requires information about the participants [44, 92]. *Requirement:* The affiliations of the different participants and multiple market sides should be represented and linked to participant profiles to support and improve the matchmaking.

#### Architectural Requirement 4: Matching as a Core Value Proposition

**Fig. 5:**
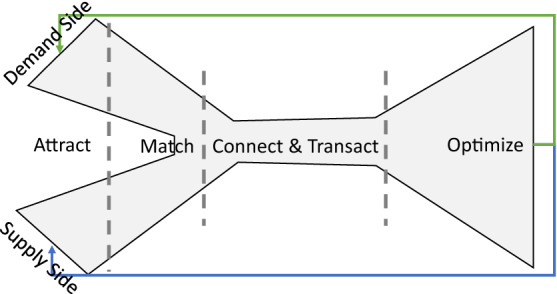
Matching market sides in e-commerce [125]

As stated, the orchestration of formerly independent market sides is the core value proposition of a digital marketplace [7, 44, 94, 95]. This involves the matching of individual participants in the market sides [83]. The matching can be described according to Reillier and Reillier [92] as a process of attracting, matching, and connecting participants to enable (retail) transactions between them. The transaction process and matching are optimized afterward [92]. The matching between supply- and demand-side participants can be illustrated in a schematic two-sided sales funnel (Fig. 5) as an extension of the e-commerce sales funnel [20]. In the attracting phase, supply- and demand-side participants are acquired and activated, while existing participants are retained as much as possible. To match both sides, the participants need to be introduced to each other, considering their characteristics captured within the participants’ affiliation with the digital marketplace. The assortment of supply-side participants, has to match the purchase desire of demand-side participants and the digital marketplace should provide appropriate matching partners [44]. Next, both participants need to be connected to execute a retail transaction that can be coordinated by the marketplace owner. Finally, the transaction is optimized in order to transact additional articles within this matched pair or derive insights for further transactions between other participants [20, 92]. *Requirement:* The matching process needs to be supported by, business and application services.

#### Architectural Requirement 5: Diversity of Services

Digital marketplaces differ in terms of the type, scope, and coverage of services offered by the marketplace owner [126]. When marketplace owners add additional services, they enhance their core value proposition. The degree of additional services offered by a digital marketplace varies on a continuum from passive matching (e.g., eBay classifieds) to full service offerings with sales processing, fulfillment services, and training services (e.g., Amazon) [117, 126]. Regarding the main bridging function [76], a substantial amount may be performed by other ecosystem participants surrounding the focal marketplace or the marketplace owner depending on the degree of centralization of the ecosystem [63, 117]. As digital marketplaces typically mature by offering additional services (e.g., Amazon and eBay) [92], the retail information system should be flexible to support the integration of additional services performed by the digital marketplace. A modular design also supports the service continuum of digital marketplaces ranging from pure matchmaking to innovative marketplaces. *Requirement:* The reference architecture should be defined in a flexible and modular way, so it supports the development and integration of services that are not yet part of the business model but are likely to be integrated in the continuing evolution of the digital marketplace.

#### Architectural Requirement 6: Innovation Platform Services

In addition to trading-related services, digital marketplaces can offer innovation platform services for marketplace participants such as access to sales data or smart product-related data or remote services not associated with the core trading business [111]. These services can also be compute power, storage, or development environments similar to the one that Amazon provides with its Web Services that originated from the variability of demand for computing resources in the e-commerce business [123]. Innovation services are the technical capabilities that enable the creation of new solutions (services or software modules) by participating third-party developers [9]. Integrating transaction and innovation services, the digital marketplace resembles an integrated platform [44]. The power of innovation platforms rests on their architectural modularity [12, 111], catalyzing the re-configurability of technical and organizational components to accelerate generativity and value creation. The components of single modules are strongly interconnected but weakly connected with the central platform through technical boundary resources [12, 110]. External modules make use of technical boundary resources provided by the innovation platform [38, 51]. *Requirement:* The reference architecture should include these innovation platform services and respective boundary resources to enable developers to exploit the offered services.

#### Architectural Requirement 7: Smart Service Provision

Digital marketplaces not only need to deal with physical and virtual products from various third-party sellers but also need to handle data generated by the continuous hybridization of physical products [100]. The augmentation of physical products with sensors, actuators, and software allows previously passive things to interact with their environment in Internet of Things scenarios [72, 90]. Data generated by these smart products can be exploited by marketplace owners to offer additional smart services to ecosystem participants that provide value to the customer and the service provider [18]. Thus, the digital marketplace needs to manage a variety of smart products; collect, aggregate, and analyze the data generated; and enable the creation and provision of smart services that exploit the data generated to create value for a customer. *Requirement:* The reference architecture should support the management of smart products, data aggregation and analysis, and provision of smart services.

#### Architectural Requirement 8: Aggregated Assortment

Digital marketplaces aggregate a digital representation of the diverse assortment of articles offered by supply-side participants. The assortment can be described as the periphery of the digital marketplace, while the core is the digital marketplace itself offering services to supply- and demand-side participants as described analogously in the platform literature [13, 106]. From the customer perspective, digital marketplaces “resemble retail agglomerations” [59] integrating the range of articles of participating suppliers, retailers, and wholesalers through a single digital channel [109]. Thus, digital marketplaces further reduce transaction costs [120] for participants as a variety of articles can be sold or purchased via a single touchpoint with a consistent user experience. Digital marketplaces also reduce the number of intermediaries that participate in a single customer journey and provide a consistent user interface [38, 51]. e-Commerce in general and the aggregation of the individual assortments of various supply-side participants require a digital representation of the articles within the assortment [112]. *Requirement:* The reference architecture should include a flexible model of the article master data to allow the aggregation of the assortment.

#### Architectural Requirement 9: Sophisticated Boundary Resources

**Fig. 6:**
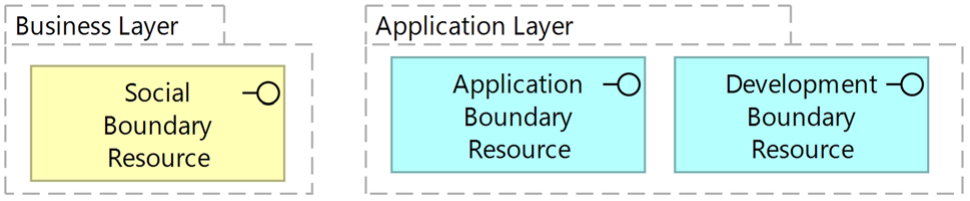
Overview of types of boundary resources for digital marketplaces [33]

The establishment of a digital marketplace involves the provision of dedicated boundary resources so that different types of participants from different markets can connect [124]. Designing boundary resources requires considering a variety of different applications. While Supply-side participants need an interface to upload their assortment to the marketplace, and service providers need interfaces to offer additional (product-related) services. External application developers use a development environment, standard system architecture, or interface descriptions [33]. The marketplace owner needs to open its retail information systems to other participants [40]. Although boundary resources allow access to core modules of the marketplace, they also act as a control mechanism, allowing marketplace owners to manage the infrastructure based on the strategy pursued, which increases the chances of achieving market leadership [38, 51]. Boundary resources represent a dimension of governance, defining the boundaries between the marketplace owner and the community of external participants, thus facilitating the realization of strategically relevant decisions about ownership, entry into new markets, or community building [33, 46, 62]. Dal Bianco et al. [33] differentiate application, development, and social boundary resources (Fig. 6). Social boundary resources are used for knowledge transfer, development boundary resources for supporting application development, and application boundary resources for enabling interaction with a focal platform. Application boundary resources (APIs, libraries, etc.) are defined as the minimum required for a software ecosystem to be viable. In contrast, development and social boundary resources increase the attractiveness of the ecosystem from the developers’ perspective [33]. *Requirement:* The reference architecture should propose sophisticated boundary resources to connect all relevant participants with a focal retailer’s information system on the business and application layers [124].

## Research Approach

**Fig. 7:**
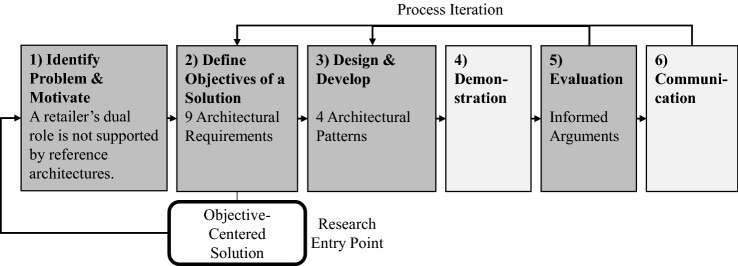
Design science research approach [89]

This publication aims to develop architectural patterns for retail information systems supporting the orchestration of multiple market sides, different types of participants in e-commerce, and innovation platform services. We applied a design science research approach as proposed by Peffers et al. [89] with an emphasis on problem identification, objectives, and solution design, presented in Fig. 7. The objective-centered process starts with the problem (1) stated in the introduction. The objectives of the artifact (2) to be developed are the architectural requirements already derived in “Digital Marketplaces”. Exemplary architectural patterns addressing the objectives (3) are designed and demonstrated (4) as an extension the shortcomings of existing reference architectures. They (5) are evaluated based on informed arguments in the discussion section [66].

The outcome of the artifact design is a model [79] or more particular (parts of) an architecture [113]. Developed as “meta-artifacts” [68], the architectural patterns represent a “general solution concept” [114] that is applicable to a class of problems when instantiated in the context of electronic retail [79]. The architectural patterns (artifacts) are a new solution to a known problem and thus resemble an improvement [53]. These patterns can describe major tasks of a digital marketplace and present them in a formal and understandable manner, applying a highly regarded enterprise architecture modeling language (i.e., ArchiMate). ArchiMate is part of The Open Group Architecture Framework (TOGAF) for the development, planning, implementation, and maintenance of enterprise architectures [125]. ArchiMate follows the service-oriented paradigm and is structured along the business, application, and infrastructure layers [125]. For this paper, we focus on modeling the business layer with related actors and processes and the application layer with its functions and services. A design science research project should go through three cycles [65]. The focus of our overarching research project is the development and evaluation of a reference architecture supporting digital marketplaces in e-commerce with transaction and innovation functions [45]; this design cycle presents architectural patterns pivotal to marketplace business models in e-commerce. Our research approach can be summarized as follows: First, we derive architectural requirements based on a previous literature analysis as presented in “Digital Marketplaces” (the rigor cycle). Second, we develop conceptual architectural patterns as general solution concepts for these requirements [69]. Thus, we develop domain-specific architectural patterns as building blocks of an overarching reference architecture based on literature [5, 48]. This design cycle focuses on deriving architectural patterns from the rigor cycle and modeling them in ArchiMate. For a future relevance cycle, we will conduct interviews with IT architects and responsible IT staff architecting (parts of) an organization’s (information systems) architecture. The retailer’s dual role and additional innovation services [50, 111] create additional requirements for retail information systems that need to be reflected in reference architectures for e-commerce. In the following section, we depict four pivotal architectural patterns for the fulfillment of the introduced architectural requirements.

## Architectural Patterns

### Pattern 1: Matching of Participants

**Fig. 8:**
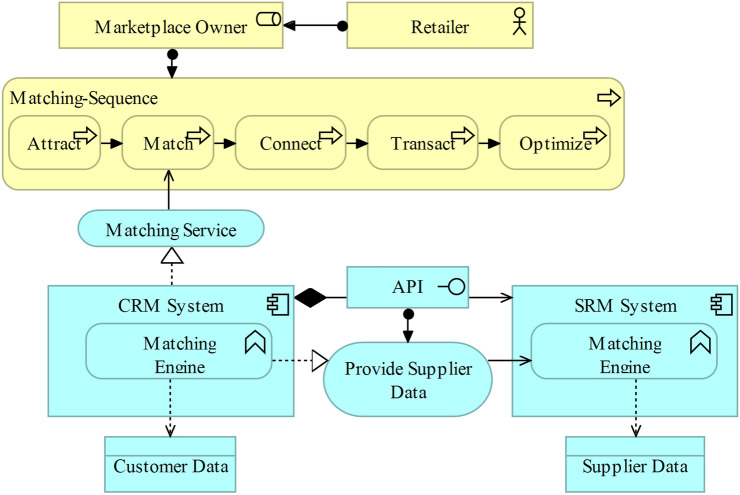
Matching as a core value proposition [125]

The first exemplary architectural pattern addressing AR5 is concerned with the matchmaking between participants from different market sides as the core value proposition of a digital marketplace [7, 27]. The matching sequence, as illustrated in Fig. 8, is executed by the retailer or wholesaler in its role as marketplace owner. The matching process is embedded in the matching sequence as proposed by Reillier and Reillier [92] and introduced in “Digital Marketplaces”. After supply- and demand-side participants are attracted,, participants from independent markets need to be matched. In this context, a customer’s desire usually leads to a product search either via search query or category search [74]. Based on the customer’s preferences stored in the customer data, the matching engine situated in the customer and supplier relationship management systems calculates the order of the listed assortment. Thus, the relevance regarding the search term is not the only factor when articles are listed as a result of the customer’s query; the preferred supplier may also be considered. Matching participants from the supply and demand sides of the digital marketplace requires an interface between these independent systems to exchange supplier and customer data relevant for the matching. The listing of the assortment is an important internal driver for retailers to increase revenues in e-commerce [25]. In digital marketplaces, the product listing is even further complicated by the retailer’s dual role, causing the question whether to emphasize products from the owner’s own assortment or from another ecosystem participant’s assortment. A higher priority for the owner’s assortment cannot be implemented because of antitrust law considerations [126]. The matching can be initiated proactively to stimulate a customer’s desire (e.g., customized newsletters, social media marketing or search engine advertising). After a match has been created successfully, the retail transaction can be executed. To optimize the matching sequence, the supplier and customer data are enriched with information derived from a previously executed transaction, and other demand-side participants in the same cluster may be notified about the previous transaction.

### Pattern 2: Innovation Services

**Fig. 9:**
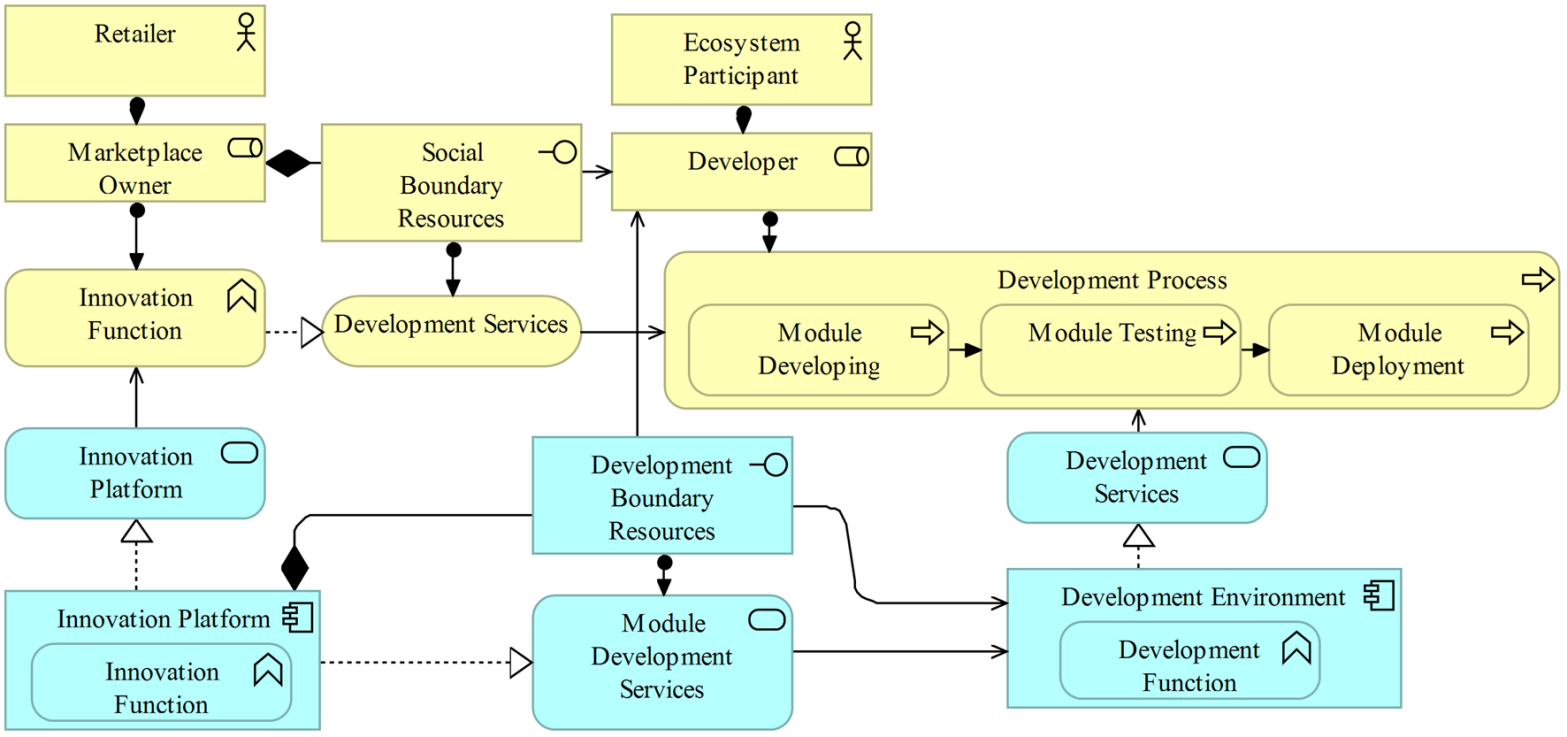
Innovation platform [125]

The second exemplary pattern addresses AR6. This conceptual pattern emphasizes the integration of innovation platform services (Fig. 9) in a digital marketplace [45, 111]. Offering innovation services [110] focusing on the development of additional modules or apps requires opening the retail information system and supporting infrastructure for third-party developers by implementing applications (e.g., APIs and SDKs) and providing social (e.g., documentation and technical support) boundary resources [38, 51]. External modules are developed using technical boundary resources provided by the innovation platform in the form of API services [38, 51].

The openness of an innovation platform defines which platform services and components from the application and infrastructure layers can be used, modified, and extended by third-party developers [122]. Openness is usually defined by the scope and richness of the interfaces offered by the platform owner [42]. The development environment can also be operated by the marketplace owner depending on the degree of openness and the boundary resources provided. Third-party developers implement additional modules such as shop themes, interfaces with other digital platforms, or feature add-ins. Although innovation platforms usually exploit economies of scale and scope with increasing efficiency and increased product variety through re-usability and reconfiguration of modules or services, they may utilize additional economic effects as the center of a broader innovation ecosystem in which they may also establish digital marketplaces [22, 32, 49]. The development process consists of, among others, processes for developing, testing, and deploying the modules. The integration of innovation services propels the development to a hyper-scaling platform [34]. Retailers increasingly establish innovation platforms and provide them to competitors to create an integrated digital business ecosystem. One example is REWE, with its subsidiaries commercetools and fulfillmenttools [29].

### Pattern 3: Boundary Resources

**Fig. 10:**
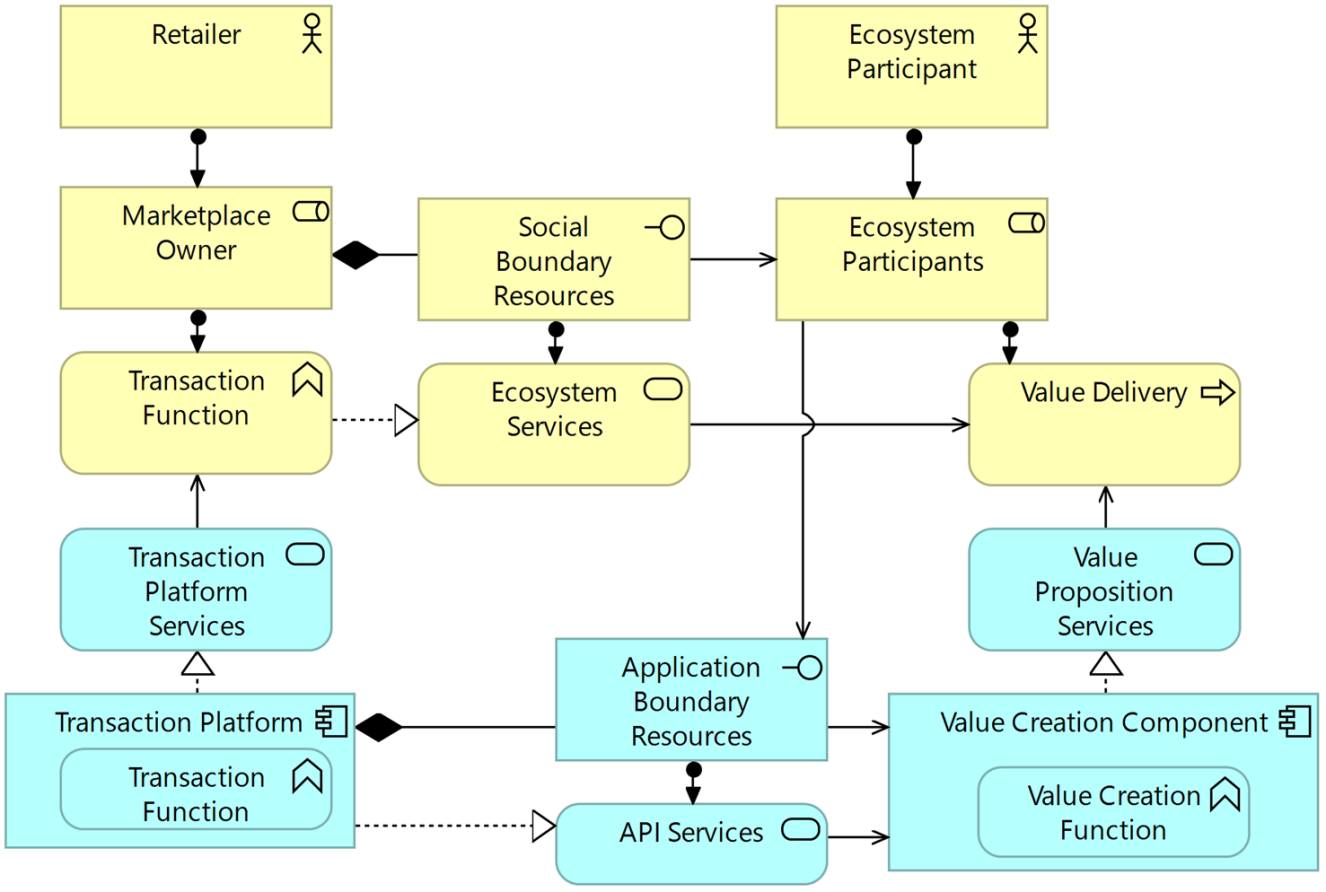
Boundary resources of a digital marketplace

The development boundary resources have been introduced in the context of the innovation platform for the development of external modules by third-party developers (Fig. 9). In this subsection, we are specifically concerned with application boundaries, opening the focal marketplace to a variety of participants (Fig. 10). These boundary resources resemble interfaces for the participants’ information systems to connect with the digital marketplace to exchange information on articles, delivery status, or customer inquiries. The ecosystem participants may involve, among others, manufacturers, wholesalers, retailers, logistic service providers, opinion researchers, content providers, or advertising partners [21, 101]. The various participants have diverging requirements regarding the interfaces offered. While a wholesaler sells merchandise via the marketplace and needs to exchange article- and customer-related data, a logistic service provider sends delivery updates and requires parcel information. In addition, the timeliness of the information varies: the description of an article is updated rarely by a content service provider, but delivery updates and payment information are sent more frequently. The application boundary resources are offered by a retailer in its role as the marketplace owner in the transaction platform. They realize application programming interfaces (APIs) that can be used by an ecosystem participant’s information systems. Each application boundary resource is augmented by dedicated social boundary resources, such as documentation, training, or guidelines [33]. This should guarantee the correct use of APIs by external application systems. There can also be social boundary resources such as general support that are not directly linked to a specific API.

### Pattern 4: Aggregated Assortment

**Fig. 11:**
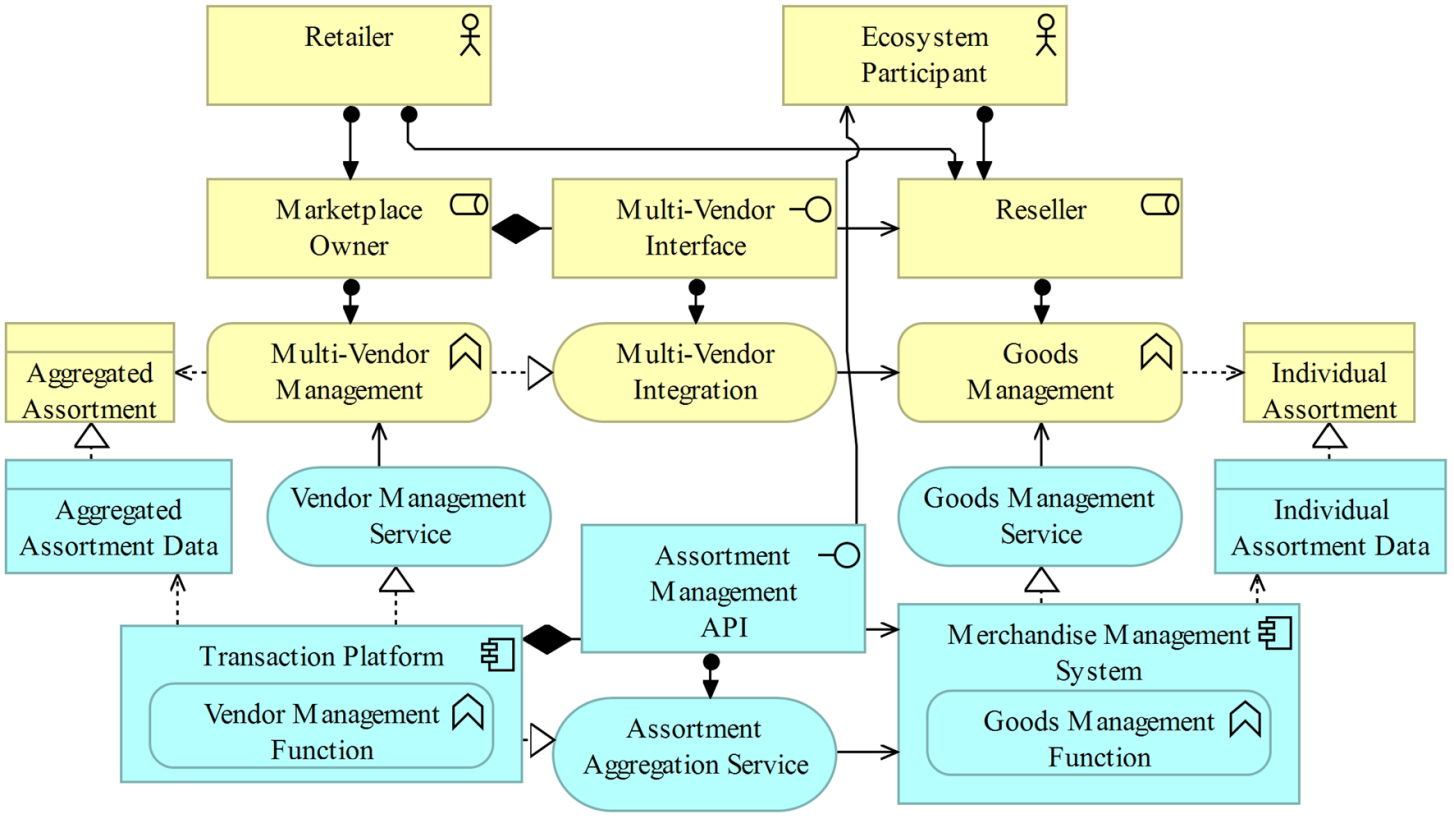
Aggregated assortment of a digital marketplace [125]

A conceptual solution for AR8 is presented in the third exemplary architectural pattern (Fig. 11). This conceptual pattern deals with the aggregation of the individual assortments of the different supply-side participants [109].

is managed by the retailer in its role as the marketplace owner. The marketplace owner aggregates the assortment of all supply-side participants in reseller mode. This may also include the retailer itself (i.e., a dual role) [56]. While a multi-vendor integration connects the supply-side participants on the business layer to the marketplace, the technical integration between the transaction platform and the merchandise management system of the reseller is realized by the assortment aggregation service on the application layer. This service provides an assortment management API connecting the systems of the marketplace owner and the supply-side participants [51]. Following the introduced concept of boundary resources in platform-centered ecosystems, the multi-vendor interface is depicted as a social boundary resource on the business layer, while the assortment management API is modeled as an application boundary resource that acts as an intermediary between the transaction platform and a third-party retailer’s merchandise management system on the application layer. For a retailer with a dual role, this means the listing of its own assortment in the marketplace. The assortment of each reseller is stored in an individual assortment business object accounting for possible additional sales channels of the reseller. The aggregated assortment is also captured by the marketplace owner in a business object realized by a data object. The data objects are managed by the transaction platform (for the marketplace owner) and the merchandise management system (for the reseller).

## Discussion

In this research paper, we present four exemplary architectural patterns that are pivotal for a retailer’s dual role in digital marketplaces. They are developed based on nine architectural requirements derived from the literature on e-commerce in general and digital marketplaces in particular. Although there could be more than one digital marketplace for a specific e-commerce sector [103], winner-takes-all tendencies and strong network effects of incumbents limit the existing number of digital marketplaces in a particular domain to only one or a few [42, 81]. A reference architecture or multiple architectural patterns supporting a retailer’s dual role in digital marketplaces simplify the assessment of the retail information system before the transition to a digital marketplace. Thus, the capabilities of the information systems for the marketplace transformation can be assessed more accurately and may decrease the possibility of failure during the establishment of the digital marketplace. Several digital marketplaces failed to establish successful digital marketplaces (e.g., zapatos, jet.com, and Rakuten) either because of the number of participants was small or because of information systems problems [126]. The domain-specific reference architecture can also be used to identify potential gaps within the retailer’s retail information system and reduce the overall time to market [4]. Moreover, a reference architecture should be augmented by a dedicated procedure for implementing the reference architecture in a specific company [102]. As reference architectures are designed to be reusable in various companies, they contain abstract descriptions of processes or functions and often provide a predefined process or component alternatives. Thus, the architectures need to be instantiated for the context of a particular company [102]. These instantiations can result in significant differences across companies (e.g., the matching process at Amazon and Otto Marketplace). An example of such a procedure is the Architecture Development Method as a major building block of TOGAF [125]. A dedicated implementation procedure can be an additional requirement for the support of a retailer’s dual role in digital marketplaces in particular and for reference architectures in general.

A retailer’s or wholesaler’s dual role in a digital marketplace results in several advantages compared to other ecosystem participants (Fig. 4). Despite possible antitrust law considerations [126], the marketplace owner will be eager to prefer the offering of its own assortment to increase reseller revenues. As the marketplace owner controls the touchpoint to the customer, the owner also has information about fast-selling and profitable articles. This information can be used to adjust the assortment of the reseller role to mainly sell profitable articles and leave the long tail [80] of articles that sell slowly to other ecosystem participants. The concentration on fast-selling articles may also release storage capacity that can be offered as additional, retail-related services to ecosystem participants [126]. With additional sales information, the marketplace owner can calculate articles for which the monetization of the matchmaking (e.g., commission fees) is more profitable than selling these articles in its reseller role. Matching as the core value proposition of a digital marketplace [7, 61] relies on correct data concerning customers, suppliers, and articles. Thus, the data needs to be stored accessibly for the matching engines to provide customers with the desired products. The actual article is the customer’s focus in the e-commerce environment [55], while suppliers are excluded by the marketplace owners [82]. Nevertheless, we propose to include supplier information in the matchmaking process, as customer preferences can be matched to suppliers’ properties. Customers who care about their environment may, for example, be likely to buy articles from a supplier that can prove sustainability. Integrating additional innovation services attracts additional participants to the digital marketplace and adds more value propositions. The range and scope of modules developed by third-party developers depend on the openness allowed by the marketplace owner and the provided development boundary resources [38, 51]. These modules can be related to the bridging functions that enhance the retail transactions between supply- and demand-side participants (e.g., shop themes, and vendor management) or go beyond retail-centered purposes. Amazon is a major example of the wide range of external modules with its Web Services stemming from the usage of unused computational power from retail activities [123]. Thus, the modeled innovation platform and development environment components are generic enough to cope with the whole continuum of external modules. The innovation pattern should be instantiated according to the intention of a specific marketplace owner. Additional types of participants and markets are attracted to a digital marketplace by opening the marketplace and offering a set of sophisticated boundary resources for each type. The openness or the degree to which a marketplace should be opened to ecosystem participants compared to proprietary parts is a topic often discussed in literature [17, 40, 88, 122, 126]. A marketplace owner needs to balance the degree of openness with the risk of losing control over its own marketplace and the surrounding ecosystem [31, 63]. Participants are likely to join an ecosystem via a marketplace creating additional focal closures and propelling network effects only if participation is valuable for them [104]. Thus, the matchmaking also needs to take into account these additional types of participants. Digital marketplaces aggregate the assortment of several supply-side participants and require a data model for the articles capable of storing much unstructured data (image, video, exploded-view drawings, spare parts with historical data, etc.). The data model must be designed to be flexible so that it is suitable for different product categories agglomerating the diverse assortments of a number of participants from independent markets [43, 73]. This may lead to a decoupling of the master data storage of a transaction processing system (e.g., enterprise resource planning) from the transaction platform and the system for product information management. This is mainly because not all articles or services of a digital marketplace can be kept on a transaction platform with all available data. While the transaction platform requires high-resolution images for digital representation in the e-commerce environment, the enterprise resource planning system is mainly concerned with financial and inventory data. However, the degree to which article data is stored on the transaction platform and provided by an additional product information system depends on the specific environment of the retailer. The product information system is not modeled in pattern 3 for reasons of graphical simplification. These architectural patterns from the first design iteration should be further evaluated with practitioner insights and aggregated to an overall reference architecture for the information system supporting a retailer’s dual role in digital marketplaces.

Aggregating transaction and innovation services, the digital marketplace forms an integrated platform [44]. The transaction platform is included in pattern 4, while the innovation platform is part of pattern 2. An integrated platform is likely to become a hyper-scaling platform that quickly achieves critical mass and shaping industries [34]. Based on our research-based requirements and patterns, we analyzed an initial sample of existing reference architectures from the e-commerce context. The preliminary sample consists of seven reference architectures-the electronic market architecture GEMS [2], ECOMOD [39], e-ZOCO architecture [24], e-commerce service composition platform (EC-SCP) [26], e-commerce reference architecture [10], integrated architecture for e-commerce [116], and next-generation e-commerce platform (NGECP) [67]-for this analysis. The initial analysis is summarized in Table 2 in chronological order of the newest reference architecture release (architectural requirement fulfilled: X; architectural requirement partially fulfilled: (X); architectural requirement not fulfilled: -).

**Table 2:** Preliminary reference architecture analysis

Reference architecture	1	2	3	4	5	6	7	8	9
GEMS [2]	–	–	x	x	–	–	–	–	–
ECOMOD [39]	–	–	(x)	–	(x)	–	–	–	–
e-ZOCO Architecture [24]	–	–	(x)	(x)	x	–	(x)	x	(x)
EC-SCP [26]	–	x	x	x	–	–	–	–	–
e-Commerce reference architecture [11]	–	–	(x)	–	x	–	–	–	–
Integrated architecture [116]	–	x	(x)	–	x	x	(x)	x	–
NGECP [67]	–	x	x	x	x	–	–	x	–

Applying our architectural requirements and developed architectural patterns to selected reference architectures revealed that none of the analyzed reference architectures for e-commerce fully supports the requirements of digital marketplaces and a retailer’s dual role. The NGECP fulfills five of the nine architectural requirements resulting from a retailer’s dual role [67]. A retailer’s dual role was not addressed by any of the analyzed reference architectures. The matching process requirement, the innovation platform services requirement, and the aggregated assortment requirement were also underrepresented in the literature sample (Table 2). The peculiarities of e-commerce and digital marketplaces require adaptations and enhancements of existing reference architectures. De Reuver et al. [93] encourage researchers to conceptualize platforms in different industries such as e-commerce. Therefore, we call for the development of reference architectures supporting digital marketplaces in general and in retail in particular.

This research paper also has its limitations. The developed architectural requirements are neither comprehensive nor complete. ARs should also be derived from practitioner sources (e.g., interviews and architecture documents) in an additional relevance cycle for a more sophisticated analysis. We plan to derive additional architectural requirements and evaluate the seven architectural requirements as well as the three architectural patterns developed by conducting interviews with IT architects at retailers that operate digital marketplaces. In addition, the developed architectural patterns must be demonstrated in specific IS implementations in the digital marketplace context. Therefore, an avenue for future research might be the implementation and evaluation in e-commerce contexts and the discussion of these patterns with practitioners (e.g., enterprise architects). Although we claimed that the architectural requirements are derived from our literature analysis and make up the sole foundation for the developed architectural patterns, we need to acknowledge that the architectural requirements are biased from our own understanding of the meta-problem. We incorporated our understanding of the retail-specific problem and the retailer’s dual role in digital marketplaces that leads to interpretations regarding the architectural requirements and architectural patterns [69]. Moreover, the chosen enterprise architecture modeling language for developing the architectural patterns leads to an implicit decision for a service-oriented architecture design, as this is inherent to this language connecting actors as well as business, application, and infrastructure layers that use the services [125]. Although service orientation is a well-regarded paradigm, it can at least be questioned whether it is the best approach for modeling the information systems architecture of a digital marketplace with the focus on the retailer’s dual role.

## Conclusion

The main contribution of this research paper is the determination of a retailer’s possible dual role in digital marketplaces. We derive nine architectural requirements resulting from a retailer’s dual role (dual role, additional participants, affiliation, matchmaking, variety of services, innovation services, smart services, aggregated assortment, and boundary resources) for a retail information system. These requirements resemble a class of problems relevant for digital marketplaces in e-commerce. In addition, we propose four architectural patterns (matchmaking for participants, innovation platform services, boundary resources, and aggregated assortment) as a conceptional solution to selected requirements. These architectural patterns were developed based on the literature and can be applied to analyze existing reference architecture toward the fulfillment of these requirements. The patterns resemble buildings blocks of a meta-model as a reference architecture for the retail domain. A preliminary analysis of existing reference architectures for e-commerce showed that a retailer’s dual role in digital marketplaces is not fully supported. Future research can analyze additional (scientific and practice) concrete architectures and reference architectures for the fulfillment of the requirements and patterns. The architectural patterns may also be improved by consolidating domain knowledge such as company-specific architectures and conducting interviews with information systems architects. Another important avenue for future research may be extending the range of architectural patterns and orchestrating them to a complete reference architecture that includes additional architecture layers. To demonstrate and evaluate the presented and additional patterns, they can be implemented in a concrete or experimental system as proof of concept.

## References

[1] Abhishek V, Jerath K, Zhang ZJ (2016). Agency selling or reselling? Channel structures in electronic retailing. Manag Sci.

[2] Albers M, Jonker CM, Karami M, Treur J (2004). Agent models and different user ontologies for an electronic market place. Knowl Inf Syst.

[3] Angelov S, Grefen P, Greefhorst D. A classification of software reference architectures: analyzing their success and effectiveness. In: 2009 Joint working IEEE/IFIP conference on software architecture and European conference on software architecture. IEEE; 2009. p. 141–50.

[4] Angelov S, Grefen P, Greefhorst D (2012). A framework for analysis and design of software reference architectures. Inf Softw Technol.

[5] Angelov S, Trienekens JJM, Grefen P. Towards a method for the evaluation of reference architectures: experiences from a case. In: Morrison R, Balasubramaniam D, Falkner K, editors. Proceedings of the European conference on software architecture (ECSA2008). Paphos: Springer; 2008;225–40.

[6] APQC: American Productivity & Quality Center Process Classification Framework (PCF)—Retail PCF. 2019. https://www.apqc.org/resource-library/resource-listing/apqc-process-classification-framework-pcf-retail-pcf-pdf-version. Accessed 24 Nov 2019.

[7] Armstrong M (2006). Competition in two-sided markets. RAND J Econ.

[8] Armstrong M, Wright J (2007). Two-sided markets, competitive bottlenecks and exclusive contracts. Econ Theory.

[9] Asadullah A, Faik I, Kankanhalli A. Digital platforms: a review and future directions. In: Proceedings of the 22nd Pacific Asia conference on information systems (PACIS 2018). Yokohama, Japan; 2018. p. 248–62.

[10] Aulkemeier F, Paramartha MA, Iacob ME, van Hillegersberg J (2016). A pluggable service platform architecture for e-commerce. Inf Syst e-Bus Manag.

[11] Aulkemeier F, Schramm M, Iacob ME, van Hillegersberg J (2016). A service-oriented e-commerce reference architecture. J Theor Appl Electron Commer Res.

[12] Baldwin CY, Clark KB (2000). Design rules: the power of modularity.

[13] Baldwin CY, Woodard JC, Gawer A (2009). The architecture of platforms: a unified view. Platforms, markets and innovation.

[14] Barth K, Hartmann M, Schröder H. Betriebswirtschaftslehre des Handels, vol. 7. überar. Wiesbaden: Springer Gabler; 2015.

[15] Bass L, Clements P, Kazman R (2003). Software architecture in practice.

[16] Becker J, Schütte R. Handelsinformationssysteme : domänenorientierte Einführung in die Wirtschaftsinformatik, 2. vollst ed. Frankfurt a. M.: Redline Wirtschaft; 2004.

[17] Benlian A, Hilkert D, Hess T (2015). How open is this platform? The meaning and measurement of platform openness from the complementors’ perspective. J Inf Technol.

[18] Beverungen D, Müller O, Matzner M, Mendling J, vom Brocke J (2019). Conceptualizing smart service systems. Electron Mark.

[19] Bhatti A, Akram H, Basit HM, Khan AU, Naqvi RSM, Bilal M (2020). E-commerce trends during COVID-19 pandemic. Int J Future Gener Commun Netw.

[20] Blank S, Dorf B (2012). The startup owner’s manual: the step-by-step guide for building a great company.

[21] Böttcher T, Rickling L, Gmelch K, Weking J, Krcmar H. Towards the digital self-renewal of retail: the generic ecosystem of the retail industry. In: Proceedings of the 16th Internationale Tagung Wirtschaftsinformatik (WI2021). Deutschland: Essen; 2021. p. 1–9.

[22] Buxmann P, Hess T (2015). Die Softwareindustrie: Ökonomische Prinzipien, Strategien.

[23] Caillaud B, Jullien B. Chicken & egg: competition among intermediation service providers. RAND J Econ. 2003;34(2):309–28.

[24] Castro-Schez JJ, Miguel R, Vallejo D, Herrera V (2010). A multi-agent architecture to support B2C e-Marketplaces: the e-ZOCO case study. Internet Res.

[25] Chen YK, Chiu FR, Yang CJ. An optimization model for product placement on product listing pages. Adv Oper Res. 2014;2014:1–9.

[26] Chi J, Yin C, Song M, Song J, Zhan X (2010). Generic service composition platform for pervasive E-Commerce. Wirel Commun Mob Comput.

[27] Choudary S (2015). Platform scale. How a new breed of startups is building large empires with minimum investment.

[28] Cloutier R, Muller G, Verma D, Nilchiani R, Hole E, Bone M (2010). The concept of reference architectures. Syst Eng.

[29] Commercetools: Lösungen für den digitalen Handel von morgen; 2020.

[30] Corallo A, Corallo A, Passiante G, Prencipe A (2007). The business ecosystem as a multiple dynamic network. The digital business ecosystem.

[31] Croitor E, Adam M. Perceived input control on digital platforms: an empirical investigation. In: Proceedings of the European Conference on Information Systems (ECIS 2020); 2020. Online Event.

[32] Cusumano MA, Gawer A (2002). The elements of platform leadership. MIT Sloan Manag Rev.

[33] Dal Bianco V, Myllärniemi V, Komssi M, Raatikainen M. The role of platform boundary resources in software ecosystems: a case study. In: Proceedings of the 11th working IEEE/IFIP conference on software architecture (WICSA22014). Sydney, Australia; 2014. p. 11–20.

[34] Dawson A, Hirt M, Scanlan J. The economic essentials of digital strategy. A supply and demand guide to digital disruption; 2016. http://www.mckinsey.com/business-functions/strategy-and-corporate-finance/our-insights/the-economic-essentials-of-digital-strategy. Accessed 23 Oct 2020.

[35] Delteil B, Le A, Miller M. Six golden rules for ecosystem players to win in Vietnam; 2020. https://www.mckinsey.com/vn/our-insights/six-golden-rules-for-ecosystem-players-to-win-in-vietnam. Accessed 30 Jan 2021.

[36] Dietz M, Khan H, Rab I. How do companies create value from digital ecosystems? 2020. https://www.mckinsey.com/business-functions/mckinsey-digital/our-insights/how-do-companies-create-value-from-digital-ecosystems. Accessed 30 Jan 2021.

[37] Easley D, Kleinberg J (2010). Networks, crowds, and markets.

[38] Eaton B, Elaluf-Calderwood S, Sorensen C, Yoo Y (2015). Distributed tuning of boundary resources: the case of Apple’s iOS service system. MIS Q Manag Inf Syst.

[39] Ecomod: Referenzgeschäftsprozesse und Strategien im E-Commerce; 2006. https://www.wi-inf.uni-duisburg-essen.de/FGFrank/ecomod/index.php. Accessed 22 Nov 2019.

[40] Eisenmann T, Parker G, Van Alstyne M. Opening platforms: how, when and why? In: Gawer A, editor. Platforms, markets and innovation. Edward Elgar, Cheltenham LB-Eisenmann; 2009. p. 131–62.

[41] Eisenmann T, Parker G, Van Alstyne MW (2006). Strategies for two-sided markets. Harv Bus Rev.

[42] Eisenmann TR (2008). Managing proprietary and shared platforms. Calif Manag Rev.

[43] Evans DS (2011). Platform economics: essays on multi-sided businesses.

[44] Evans DS, Schmalensee R. Matchmakers. The new economics of multisided platforms. Boston: Harvard Business Review Press; 2016.

[45] Evans PC, Gawer A. The rise of the platform enterprise: a global survey (2016). https://www.thecge.net/app/uploads/2016/01/PDF-WEB-Platform-Survey_01_12.pdf. Accessed 28 Dec 2020.

[46] Foerderer J, Kude T, Schuetz SW, Heinzl A (2019). Knowledge boundaries in enterprise software platform development: antecedents and consequences for platform governance. Inf Syst J.

[47] Galster M. Software reference architectures: related architectural concepts and challenges. In: 1st International workshop on exploring component-based techniques for constructing reference architectures (CobRA); Montréal, Canada. 2015. p. 5–8.

[48] Galster M, Avgeriou P. Empirically-grounded reference architectures: a proposal. In: Proceedings of the joint ACM SIGSOFT conference-QoSA and ACM SIGSOFT symposium-ISARCS on quality of software architectures-QoSA and architecting critical systems (ISARCS2011); Boulder, USA. 2011. p. 153–8.

[49] Gawer A, Gawer A (2009). Platform dynamics and strategies: from products to services. Platforms, markets and innovation.

[50] Gawer A, Henderson R (2007). Platform owner entry and innovation in complementary markets: evidence from Intel. J Econ Manag Strategy.

[51] Ghazawneh A, Henfridsson O (2013). Balancing platform control and external contribution in third-party development: the boundary resources model. Inf Syst J.

[52] Giachetti RE (2010). Design of enterprise systems: theory, architecture, and methods.

[53] Gregor S, Hevner AR (2013). Positioning and presenting design science research for maximum impact. MIS Q Manag Inf Syst.

[54] Grieger M (2003). Electronic marketplaces: a literature review and a call for supply chain management research. Eur J Oper Res.

[55] Hagberg J, Sundstrom M, Egels-Zandén N (2016). The digitalization of retailing: an exploratory framework. Int J Retail Distrib Manag.

[56] Hagiu A (2007). Merchant or two-sided platform?. Rev Netw Econ.

[57] Hagiu A, Wright J (2015). Multi-sided platforms. Int J Ind Organ.

[58] Hanke J, Hauser M, Durr A, Thiesse F. Redefining the offline retail experience: designing product recommendation. In: Proceedings of the 26th European conference on information systems (ECIS2018), Portsmouth, UK; 2018. p. 1–14.

[59] Hänninen M (2018). Has digital retail won?: the effect of multi-sided platforms on the retail industry. Strateg Dir.

[60] Haucap J, Heimeshoff U. Ordnungspolitik in der digitalen Welt: Ordnungsdefizite und Lösungsansätze. In: Thieme J, Haucap J, editors. Wirtschaftspolitik im Wandel: Ordnungsdefizite und LösungsansätzeSchriften zu Ordnungsfragen der Wirtschaft 105. Oldenburg: De Gruyter; 2018. p. 79–132.

[61] Haucap J, Wenzel T. Wettbewerb im Internet: Was ist online anders als offline? Zeitschrift für Wirtschaftspolitik. 2011;60(2):200–11. internal-pdf://0916673775/Haucamp et al (2011) Wettbewerb im Internet -.pdf LB - Haucap2011.

[62] Hein A, Schreieck M, Riasanow T, Setzke DS, Wiesche M, Böhm M, Krcmar H (2020). Digital platform ecosystems. Electron Mark.

[63] Hein A, Schreieck M, Wiesche M, Krcmar H. Multiple-case analysis on governance mechanisms of multi-sided platforms. In: Nissen V, Stelzer D, Straßburger S, Fischer D, editors. Multikonferenz Wirtschaftsinformatik (MKWI) 2016; 2016. p. 1613–24. Ilmenau.

[64] Heinrich L, Stelzer D. Informationsmanagement: Grundlagen, Aufgaben. München: Methoden. Oldenbourg Wissenschaftsverlag; 2009.

[65] Hevner A, Chatterjee S. Design research in information systems. Integrated series in information systems, vol. 22. New York: Springer [u.a.]; 2010.

[66] Hevner AR, March ST, Park J, Ram S (2004). Design science in information systems research. MIS Q Manag Inf Syst.

[67] Huang Y, Chai Y, Liu Y, Shen J (2019). Architecture of next-generation e-commerce platform. Tsinghua Sci Technol.

[68] Iivari J. The IS core—VII: towards information systems as a science of meta-artifacts. Commun Assoc Inf Syst. 2003;12:568–81. 10.17705/1cais.01237

[69] Iivari J (2015). Distinguishing and contrasting two strategies for design science research. Eur J Inf Syst.

[70] Ivarsson F, Svahn F. Digital and conventional matchmaking—similarities, differences and tensions. In: Proceedings of the 53rd Hawaii international conference on system sciences (HICCS 2020), Maui, Hawaii; 2020. p. 5932–41.

[71] Kawa A, Wałȩsiak M (2019). Marketplace as a key actor in e-commerce value networks. Logforum.

[72] Knackstedt R. Fachkonzeptionelle Referenzmodellierung einer Managementunterstützung mit quantiativen und qualitativen Daten. Berlin: Methodische Konzepte zur Konstruktion und Anwendung. Logos Verlag; 2006.

[73] Kollmann T. E-Business. Grundlagen elektronischer Geschäftsprozesse in der Digitalen Wirtschaft, 7., überar edn. Wiesbaden: Springer Gabler; 2019.

[74] Kotler P, Keller KL. Marketing management, 16th edn. Boston: Pearson [u.a.]; 2016.

[75] Laudon KC, Traver CG. E-Commerce 2018: business, technology, society, vol. 14. Boston: Pearson Education; 2019.

[76] Levy M, Weitz BA, Grewal D. Retailing management, 10th. edit. ed. New York: McGraw-Hill Education; 2019.

[77] Li Q, Wang Q, Song P (2019). The effects of agency selling on reselling on hybrid retail platforms. Int J Electron Commer.

[78] Li Z, Penard T (2014). The role of quantitative and qualitative network effects in B2B platform competition. Manag Decis Econ.

[79] March ST, Smith GF (1995). Design and natural science research on information technology. Decis Support Syst.

[80] McAfee A, Brynjolfsson E. Machine, platform, crowd: harnessing our digital future. New York: W W Norton & Co Inc, LB-Mcafee; 2017.

[81] McIntyre DP, Srinivasan A (2017). Networks, platforms, and strategy: emerging views and next steps. Strateg Manag J.

[82] McKinsey: the conflicted continent: ten charts show how COVID-19 is affecting consumers in Europe (2020). https://www.mckinsey.com/business-functions/marketing-and-sales/our-insights/the-conflicted-continent-ten-charts-show-how-covid-19-is-affecting-consumers-in-europe. Accessed 30 Jan 2021.

[83] Moazed A, Johnson LN. Modern monopolies. What it takes to dominate the 21st-century economy. New York: St. Martin’s Press; 2016.

[84] Müller-Hagedorn L, Toporowski W, Zielke S. Der Handel : Grundlagen - Management - Strategien, 2. vollst ed. Stuttgart: Verlag W. Kohlhammer; 2012.

[85] Nakagawa EY, Oliveira Antonino P, Becker M. Reference architecture and product line architecture: A subtle but critical difference. In: Proceedings of the 5th European conference on software architecture (ECSA 2011), Essen, Deutschland. LNCS, vol. 6903; 2011. p. 207–11.

[86] Nicola M, Alsafi Z, Sohrabi C, Kerwan A, Al-Jabir A, Iosifidis C, Agha M, Agha R (2020). The socio-economic implications of the coronavirus pandemic (COVID-19): a review. Int J Surg.

[87] OMG: ARTS business process model for retail (2019). https://www.omg.org/retail-depository/arts-bpm/. Accessed 24 Nov 2019.

[88] Ondrus J, Gannamaneni A, Lyytinen K. The impact of openness on the market potential of multi-sided platforms: a case study of mobile payment platforms. J Inf Technol. 2015;30:260–75. internal-pdf://220.137.165.237/Ondrus et al (2015) The impact of openness on.pdf LB - Ondrus2015.

[89] Peffers K, Tuunanen T, Rothenberger MA, Chatterjee S (2007). A design science research methodology for information systems research. J Manag Inf Syst.

[90] Porter ME, Heppelmann JE. How smart, connected products are transforming competition. (Spotlight on managing the Internet of Things). Harv Bus Rev. 2014;92(11):1–23.

[91] Recker J, Lukyanenko R, Samuel BM, Castellanos A (2021). From representation to mediation: a new agenda for conceptual modeling research in a digital world. MIS Q.

[92] Reillier LC, Reillier B. Platform strategy. How to unlock the power of communities and networks to grow your business. London: Routledge; 2017.

[93] de Reuver M, Sørensen C, Basole RC (2017). The digital platform: a research agenda. J Inf Technol.

[94] Rochet J, Tirole J (2003). Platform competition in two-sided markets. J Eur Econ Assoc.

[95] Rochet J, Tirole J (2006). Two-sided markets: a progress report. RAND J Econ.

[96] Rotar A. eCommerce Report 2020 (2020). https://www.statista.com/study/42335/ecommerce-report/. Accessed 30 Jan 2021.

[97] Rudolph T, Nagengast L, Melanie B, Bouteiller D (2015). Die Nutzung mobiler Shopping Apps im Kaufprozess. Mark Rev St. Gallen.

[98] Schütte R, Dubois E, Pohl K (2017). Information systems for retail companies. International conference on advanced information systems engineering.

[99] Schütte R. Retailing in Zeiten der Digitalisierung. Die Plattformen dominieren den Wettbewerb. IM+io Best & Next Practices aus Digitalisierung | Management | Wissenschaft **Heft 4**; 2018. p. 46–51.

[100] Schütte R, Vetter T. Analyse des Digitalisierungspotentials von Handelsunternehmen. In: Gläß R, Leukert B, editors. Handel 4.0: Die Digitalisierung des Handels-Strategien, Technologien, Transformation. Berlin: Springer Gabler [u.a.]; 2016. p. 75–112.

[101] Schütte R, Wulfert T. Digital platforms and trading companies: the evolution of traditional business ecosystems to integrated digital business ecosystems. In: Baumann S, editor. Handbook on digital business ecosystems: technologies, markets, business models, management, and societal challenges, chap. 15. Cheltenham: Edward Elgar; 2022. p. 212–31.

[102] Schütte R, Wulfert T. Referenzmodelle und -architekturen: wiederverwendbare Modelle zur Unterstützung der Digitalen Transformation. In: Roth S, Corsten H, editors. Handbuch Digitalisierung. München: Vahlen; 2022. p. 1–29.

[103] Senyo PK, Liu K, Effah J (2019). Digital business ecosystem: literature review and a framework for future research. Int J Inf Manag.

[104] Shapiro C, Varian HRLBS. Information rules: a strategic guide to the network economy. Boston: Harvard Business Press; 1998.

[105] Shaw M, Garlan D. Software architecture. Perspectives on an emerging discipline. Upper Saddle River: Prentice-Hall; 1996.

[106] Staykova KS, Damsgaard J. A typology of multi-sided platforms: the core and the periphery. In: ECIS 2015 proceedings, vol. Paper; 2015. p. 174.

[107] Täuscher K, Laudien S. Understanding platform business models: a mixed methods study of marketplaces. Eur Manag J. 2018;36(3):319–29. 10.1016/j.emj.2017.06.005.

[108] Taylor RN, Medvidovic N, Dashofy EM. Software architecture. Foundations, theory and practice. Wiley, Hoboken; 2010.

[109] Teller C, Elms J (2010). Managing the attractiveness of evolved and created retail agglomerations formats. Mark Intell Plan.

[110] Tiwana A (2015). Evolutionary competition in platform ecosystems. Inf Syst Res.

[111] Tiwana A, Konsynski B, Bush AA (2010). Platform evolution: coevolution of platform architecture, governance, and environmental dynamics. Inf Syst Res.

[112] Turban E, Whiteside J, King D, Outland J. Introduction to electronic commerce and social commerce. 4th ed. Springer texts in business and economics. Cham: Springer International Publishing; 2017.

[113] Vaishnavi V, Kuechler B, Petter S. Design science research in information systems; 2019. http://www.desrist.org/design-research-in-information-systems/. Accessed 30 Jan 2021.

[114] Van Aken JE (2004). Management research based on the paradigm of the design sciences: the quest for field-tested and grounded technological rules. J Manag Stud.

[115] Van Alstyne M, Parker G, Choudary SP. 6 Reasons platforms fail. Harv Bus Rev. 2016;31(6):1–6.

[116] Vetter T, Morasch R, Heinemann G, Gehrckens HM, Täuber M, GmbH A (2019). Integrierte Plattformen im Handel. Handel mit Mehrwert.

[117] Wang S, Archer NP (2007). Electronic marketplace definition and classification: literature review and clarifications. Enterp Inf Syst.

[118] West J (2003). How open is open enough? Melding proprietary and open source platform strategies. Res Policy.

[119] Willcocks LP, Feeny D, Islei G (1997). Managing information technology as a strategic resource.

[120] Williamson OE. The economic institutions of capitalism. Firms, markets, relational contracting. New York: The Free Press/MacMillan [u.a.]; 1985.

[121] Winter R, Fischer R. Essential layers, artifacts, and dependencies of enterprise architecture. In: Proceedings of the 10th IEEE international enterprise distributed object computing conference workshops (EDOCW2006), Hong Kong, China. 2006. p. 30–8.

[122] Witte A, Zarnekow R. Is open always better?—a taxonomy-based analysis of platform ecosystems for fitness trackers. In: Multikonferenz Wirtschaftsinformatik, Lüneburg; 2018. p. 732–42.

[123] Wittig M, Wittig A, Whaley B (2016). Amazon web services in action.

[124] Wulfert T, Busch J. Reference architectures facilitating a retailer’s dual role on digital marketplaces: a literature review. In: Proceedings of the 24th international conference on enterprise information systems (ICEIS2022); Online Event. 2022.

[125] Wulfert T, Schütte R. Retailer’s dual role in digital marketplaces: towards architectural patterns for retail information systems. In: Proceedings of the 23rd international conference on enterprise information systems (ICEIS 2021); Online Event. 2021. p. 601–12.

[126] Wulfert T, Seufert S, Leyens C. Developing multi-sided markets in dynamic electronic commerce ecosystems—towards a taxonomy of digital marketplaces. In: Proceedings of the 54th Hawaii international conference on system sciences (HICSS 2021), Maui, Hawaii; 2021. p. 6133–42.

